# Supplementary material for: A Critical View of the Application of the APEX Software (Aqueous Photochemistry of Environmentally-Occurring Xenobiotics) to Predict Photoreaction Kinetics in Surface Freshwaters
Source: Molecules. 2019 Dec 18;25(1):9. doi: 10.3390/molecules25010009 (PMC7017383; doi:10.3390/molecules25010009)
Supplement: Supplementary file 1 [file molecules-25-00009-s001.zip › 3.2.4_gcc-4.4.0.zip!/3.2.4_gcc-4.4.0/3.2.4_gcc-4.4.0/doc/octave/html/interpreter/Line-series.html]

Line series - Untitled


Next: Quiver group,
Previous: Error bar series,
Up: Object Groups


---

##### 15.2.8.6 Line series

Line series objects are created by the `plot` and `plot3`
functions and are of the type `line`. The properties of the
line series with the ability to add data sources.

`color`: The RGB color or color name of the line objects. See Colors. `linewidth` `linestyle`: The line width and style of the line objects. See Line Styles. `marker` `markeredgecolor` `markerfacecolor` `markersize`: The line and fill color of the markers. See Colors. `xdata` `ydata` `zdata`: The original x, y and z data. `xdatasource` `ydatasource` `zdatasource`: Data source variables.
